# New Insights of Transcriptional Regulator AflR in Aspergillus flavus Physiology

**DOI:** 10.1128/spectrum.00791-21

**Published:** 2022-01-26

**Authors:** Peng Wang, Jia Xu, Perng-Kuang Chang, Zhemin Liu, Qing Kong

**Affiliations:** a School of Food Science and Engineering, Ocean University of China, Qingdao, Shandong, China; b Southern Regional Research Center, Agricultural Research Service, US Department of Agriculture, New Orleans, Louisiana, United States; Universidade de Sao Paulo

**Keywords:** *Aspergillus flavus*, aflatoxin, *aflR*, asexual reproduction, sclerotial development

## Abstract

Aspergillus flavus *aflR*, a gene encoding a Zn(II)_2_Cys_6_ DNA-binding domain, is an important transcriptional regulator of the aflatoxin biosynthesis gene cluster. Our previous results of Gene ontology (GO) analysis for the binding sites of AflR in A. flavus suggest that AflR may play an integrative regulatory role. In this study the Δ*aflR* and overexpression (OE) strains based on the well-established double-crossover recombinational technique were constructed to investigate the integrative function of the *aflR* gene in A. flavus. The disruption of *aflR* severely affected the aflatoxin biosynthetic pathway, resulting in a significant decrease in aflatoxin production. The aflatoxin B_1_ (AFB_1_) of the Δ*aflR* strain was 180 ng/mL and aflatoxin B_2_ (AFB_2_) was 2.95 ng/mL on YES medium for 5 days, which was 1/1,000 of that produced by the wild-type strain (WT). In addition, the Δ*aflR* strain produced relatively sparse conidia and a very small number of sclerotia. On the seventh day, the sclerotia yield on each plate of the WT and OE strains exceeded 1,000, while the sclerotial formation of the Δ*aflR* strain was not detected until 14 days. However, the biosynthesis of cyclopiazonic acid (CPA) was not affected by *aflR* gene disruption. Transcriptomic analysis of the Δ*aflR* strain grown on potato dextrose agar (PDA) plates at 0 h, 24 h, and 72 h showed that expression of clustering genes involved in the biosynthesis of aflatoxin was significantly downregulated. Meanwhile, the Δ*aflR* strain compared with the WT strain showed significant expression differences in genes involved in spore germination, sclerotial development, and carbohydrate metabolism compared to the WT. The results demonstrated that the A. flavus *aflR* gene also played a positive role in the fungal growth and development in addition to aflatoxin biosynthesis.

**IMPORTANCE** Past studies of the A. flavus *aflR* gene and its orthologues in related Aspergillus species were solely focused on their roles in secondary metabolism. In this study, we used the Δ*aflR* and OE strains to demonstrate the role of *aflR* in growth and development of A. flavus. For the first time, we confirmed that the Δ*aflR* strain also was defective in production of conidia and sclerotia, asexual propagules of A. flavus. Our transcriptomic analysis further showed that genes involved in spore germination, sclerotial development, aflatoxin biosynssssthesis, and carbohydrate metabolism exhibited significant differences in the Δ*aflR* strain compared with the WT strain. Our study indicates that AflR not only plays an important role in regulating aflatoxin synthesis but also in playing a positive role in the conidial formation and sclerotial development in A. flavus. This study reveals the critical and positive role of the *aflR* gene in fungal growth and development, and provides a theoretical basis for the genetic studies of other *aspergilli*.

## INTRODUCTION

Zinc cluster proteins are one of the largest families of transcriptional regulators in eukaryotes and play multiple functions in transcription (1, 2). Based on their unique and highly conserved amino acid sequences, eukaryotic zinc cluster proteins are classified into three major classes: Cys_2_His_2_ (C_2_H_2_), Cys_4_ (C4), and Cys_6_ (C6) (3). The protein AflR encoded by *aflR* is a binuclear cluster protein Zn(II)_2_Cys_6_ (C_6_) transcription factor of the GAL4-type family (4, 5). C_6_ proteins commonly contain two functional domains, the DNA-binding domain which includes the Zn(II)_2_Cys_6_ motif, the linker region, and downstream basic dimerization region, and the regulatory domain which is a specific transcription factor (TF) domain (6). The dimerization region, which is usually located at the C-terminus of the linker, consists of a heptapeptide repeat sequence similar to that found in the leucine zipper. The heptapeptide repeat sequence forms a highly conserved convoluted helix structure that most likely leads to dimerization and protein–protein interactions (7).

C_6_ proteins are mainly associated with genes of the utilization of carbon sources, nitrogen sources, secondary metabolism, growth and development, and play a global regulatory role in fungi (6). In Aspergillus flavus, disruption of *aswA* renders abnormalities in sclerotial development and biosynthesis of secondary metabolites (8). In Aspergillus nidulans, the zinc cluster protein SfgA negatively regulates the activator of FluG in asexual development (9). The C_6_ protein ADA-6 plays a vital role in conidial formation, sexual development, and oxidative stress in Neurospora crassa (10). C_6_ transcription factors have an important role in fungal growth, development, and secondary metabolism-related gene expression or repression. Therefore, unraveling the integrated regulatory role and mechanism of important transcription factors is essential to gain insight into the fungal growth and development and metabolic pathways research.

In A. flavus, the AflR protein binds to at least 17 genes in the aflatoxin biosynthetic cluster, leading to the activation of an enzymatic cascade reaction that results in aflatoxin biosynthesis (11–15). When the *aflR* gene was overexpressed, it resulted in higher transcript accumulation and increased aflatoxin production (16). Promoter regions of several biosynthesis genes are bound by AflR in a dimeric form with a 5′-TCG(N5)CGA-3′ binding motif. AflR also recognizes 5′-TTAGGCCTAA-3′ and 5′-TCGCAGCCCGG-3′ binding sequences (17). In A. parasiticus, AFLR transcription factor binding sites for the genes *nadA*, *hlyC*, and *niiA* were found outside the aflatoxin gene cluster providing the first evidence that genes outside the aflatoxin gene cluster may be AflR regulation (18). Kong et al. reported that the binding motif of AflR in A. flavus is 5′-CSSGGGWTCGAWCCCSSG-3′, an 18 bp palindrome sequence (19). Their ChIP-seq analysis showed that the consensus AflR binding sequences are present in 5′-NTR of 540 genes that were outside the aflatoxin biosynthesis gene cluster (19). To date, research on AflR has focused on the effects of AflR on aflatoxin biosynthesis, but the role of AflR in fungal growth and development and on genome-wide gene expression remains to be explored. This study aimed to investigate the integrated regulatory role of *aflR* in A. flavus.

We verified the effect of *aflR* gene on A. flavus conidial formation, sclerotial development, and biosynthesis of aflatoxin and cyclopiazonic acid (CPA) by examining *aflR* gene disrupted strains under different media. In addition to the effect on aflatoxin biosynthesis, *aflR* knockout significantly affected sclerotial development of A. flavus. Then, transcriptome profiles generated by RNA-seq of the Δ*aflR* mutant and wild type at 0 h, 24 h, and 72 h on potato dextrose agar (PDA) media was measured to study the effect of *aflR* on A. flavus.

## RESULTS

### Structural prediction and phylogenetic analysis of AflR in A. flavus.

**FIG 1:**
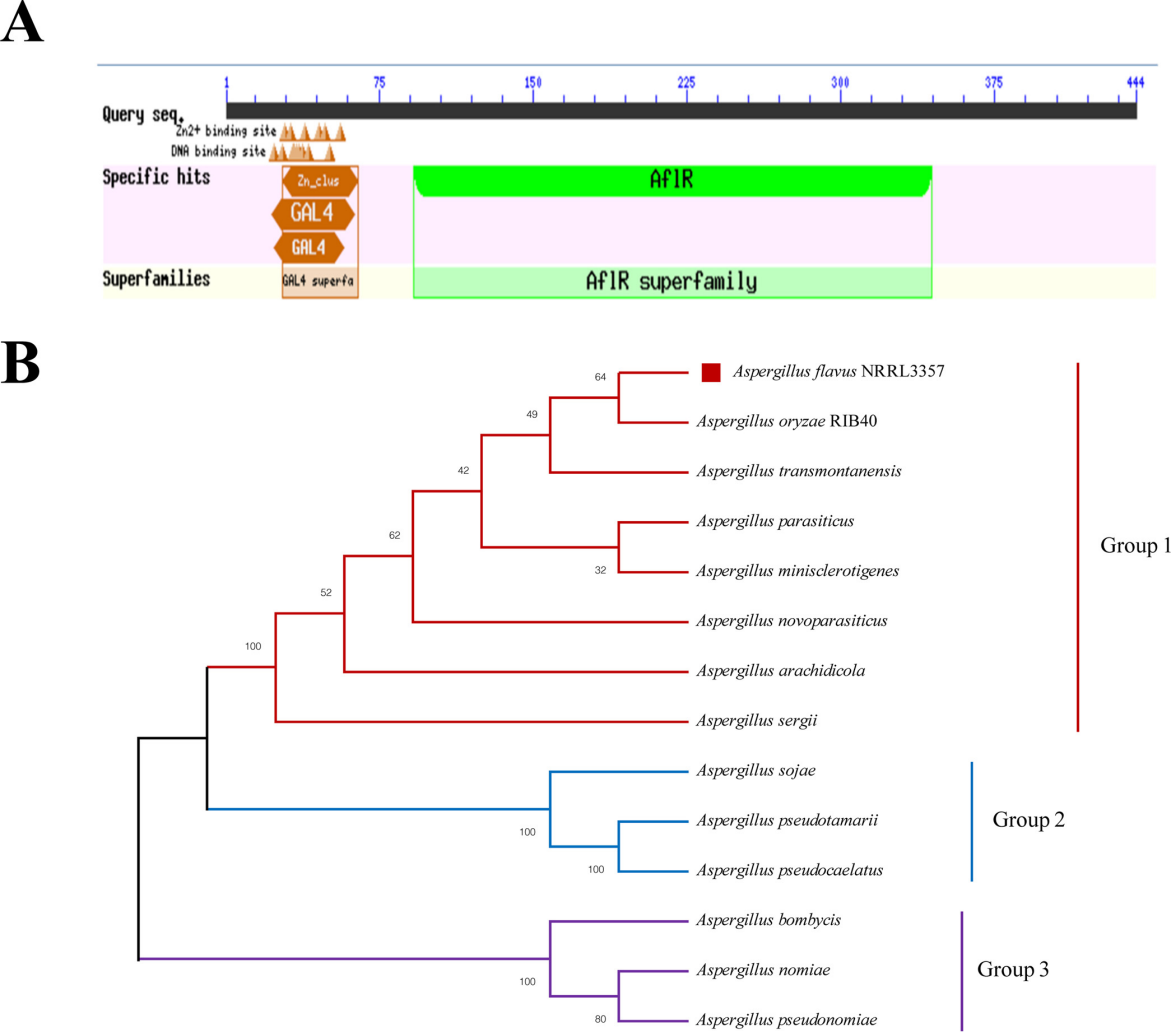
Conserved domains of the AflR protein (A) and phylogenetic analysis of the AflR proteins (B).

A. flavus NRRL3357 *aflR* gene is 1,335 bp in length. AflR contains 444 amino acids. Amino acid residues 29 to 56 of AflR form a GAL4-type binuclear zinc cluster Zn(II)_2_Cys_6_ (C6) structure (Fig. 1A). AflR and its homologs from closely related Aspergillus species were analyzed by using MEGA X, and an evolutionary tree was constructed (Fig. 1B) (20). AflR shared 99.10% and 99.32% sequence identity with those of A. oryzae and A. transmontanensis, respectively. According to the phylogenetic relationship, A. flavus is more closely related to A. oryzae than to other Aspergillus species. The *aflS* gene is adjacent to the *aflR* gene and encodes an important transcriptional regulator, which in combination with the *aflR* gene plays a role in regulating aflatoxin biosynthesis (21, 22). Therefore, in this study, we compared the full gene sequence of *aflS* by whole gene sequencing, and the sequencing comparison. Our sequencing results showed that showed that the full-length sequence of the *aflS* gene of the Δ*aflR* strain was identical to the full sequence of *aflS* gene to that of the wild-type (WT) strain (Fig. S3).

### Effect of *aflR* on mycelial growth and conidial formation.

**FIG 2:**
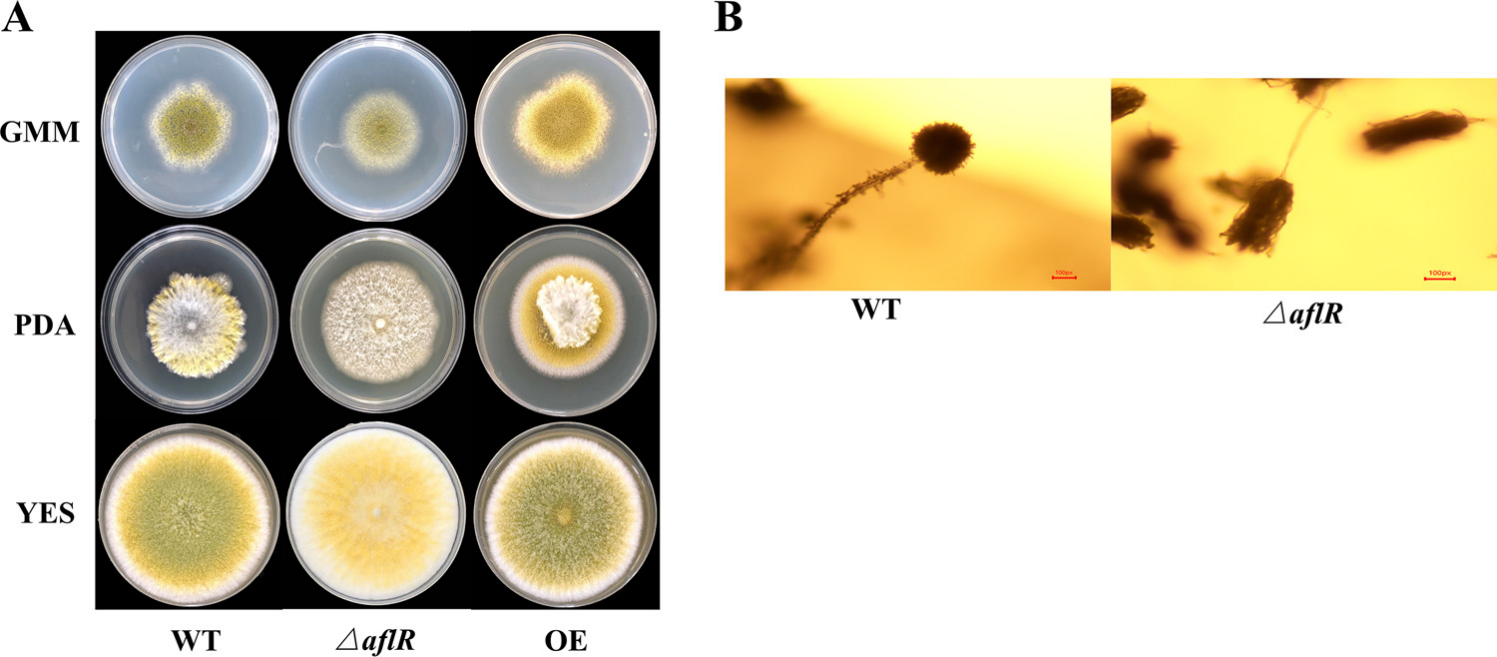
Colony morphology of the WT, *ΔaflR*, and OE strains on different media (GMM, PDA, and YES) (A) and microscopic observation of the WT and Δ*aflR* strains conidiophores at day 3 on GMM medium (10× eyepiece, 40× objective) (B).

Colony morphologies of A. flavus WT, Δ*aflR*, and overexpression (OE) strains on the three media (glucose minimal medium [GMM], PDA, and YES) were significantly different (Fig. 2A). The WT, Δ*aflR*, and OE strains on YES medium, with dense mycelia, produced large numbers of yellow-green spores. On PDA medium, they also produced dense mycelia. Their radial growth on GMM was slow and the colony diameter was small, but the OE strain showed the largest colony diameter. Compared with the WT strains, the Δ*aflR* strains had defective conidial head development. The WT strains began to form conidial heads and strong stalks at 24 h, while the Δ*aflR* strains did not begin to form scattered conidial heads and slender stalks until 30 h (Fig. S4). The WT strains had round-shaped conidial heads full of well-developed conidia, while the Δ*aflR* strain showed broom-like, elongated conidial heads with smaller conidia (Fig. 2B).

**FIG 3:**
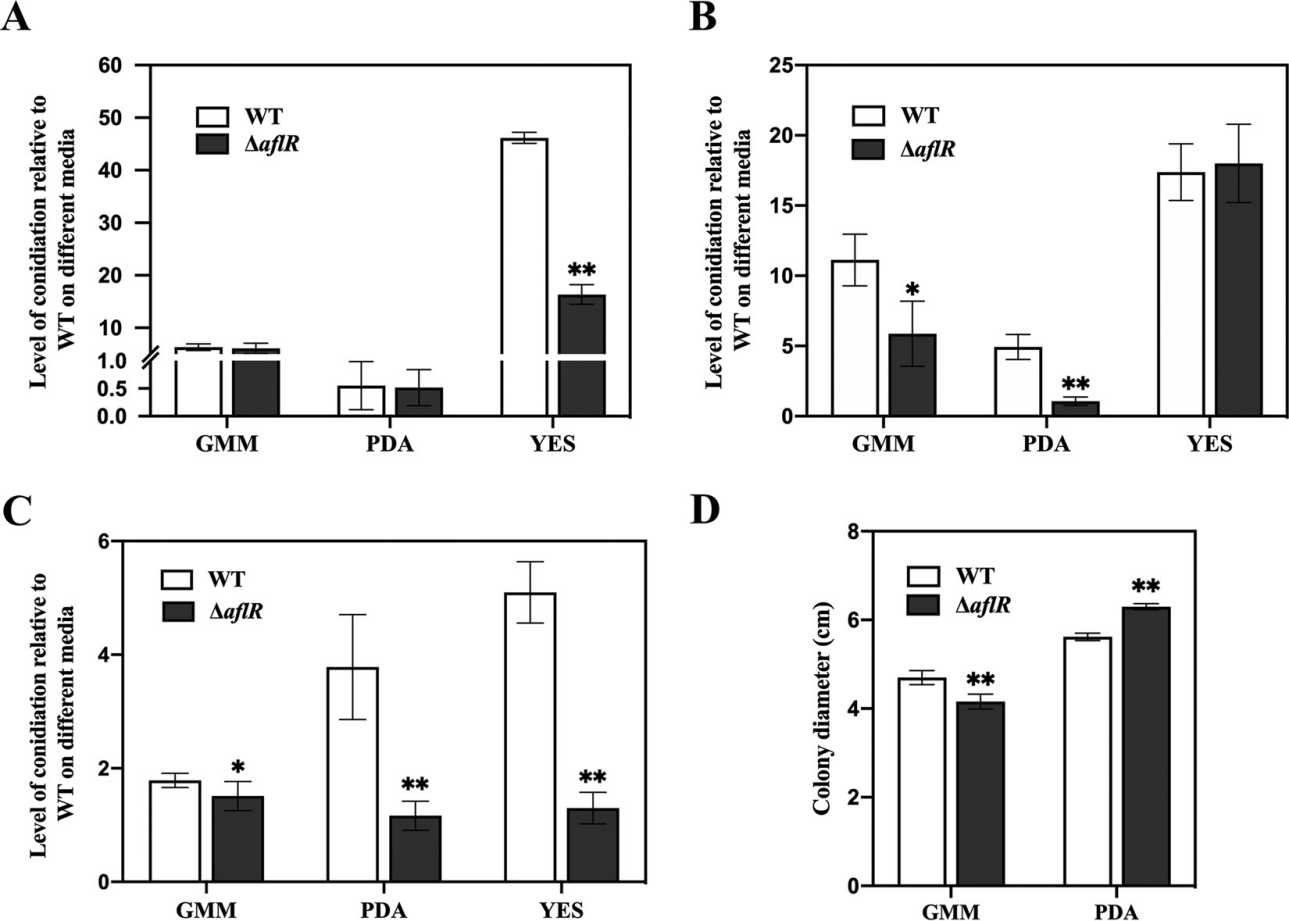
Conidial production of the WT and Δ*aflR* strains on different media at day 3 (A), day 5 (B), and day 7 (C). Plates were incubated at 30°C and circular agar plugs (1.5 cm in diameter) in triplicate were sampled from each plate and the number of conidia was counted with a hemocytometer. At day 5, the colony diameter was different between the WT and Δ*aflR* strains (D). * *P < *0.05; ** *P < *0.01.

To further investigate the effect of *aflR* on conidia formation, the conidial count was determined at different times (3, 5, and 7 days) on GMM, PDA, and YES. The Δ*aflR* strain, in general, showed a small but significant decrease in spore count compared with the WT strain especially at day 7 on all three media. Conidia of the Δ*aflR* strain were restricted, but conidial production was not completely lost. On day 3 (Fig. 3A), conidial formation of A. flavus on GMM and PDA media were at an early stage of development. Thus, there was no significant difference in conidial production between the Δ*aflR* and WT strains on GMM and PDA media, while the Δ*aflR* strain showed significantly lower spore production compared with the WT strain on YES media. The WT strain conidia on GMM and PDA media started to develop rapidly from the fifth day, while the Δ*aflR* strain conidia were relatively slow, resulting in a significant difference in conidial numbers (Fig. 3B). Compared with the WT strain, significant differences were observed on all media for the Δ*aflR* strain from day 7 (Fig. 3C). The smallest difference was observed on the GMM medium, where spore production was similar, while the largest difference was observed on the YES medium, reaching a difference of about 5-fold. Compared with the WT strain, the conidia of the OE strain increased in different degrees on three media (Fig. S5). The Δ*aflR* and WT strains showed significant differences in colony diameters on GMM and PDA media (Fig. 3D), while at 5 days on YES medium, the Δ*aflR* and WT strains had grown all over the YES plates and colony diameters could not be determined.

### Effect of *aflR* on the development of sclerotia of A. flavus.

**FIG 4:**
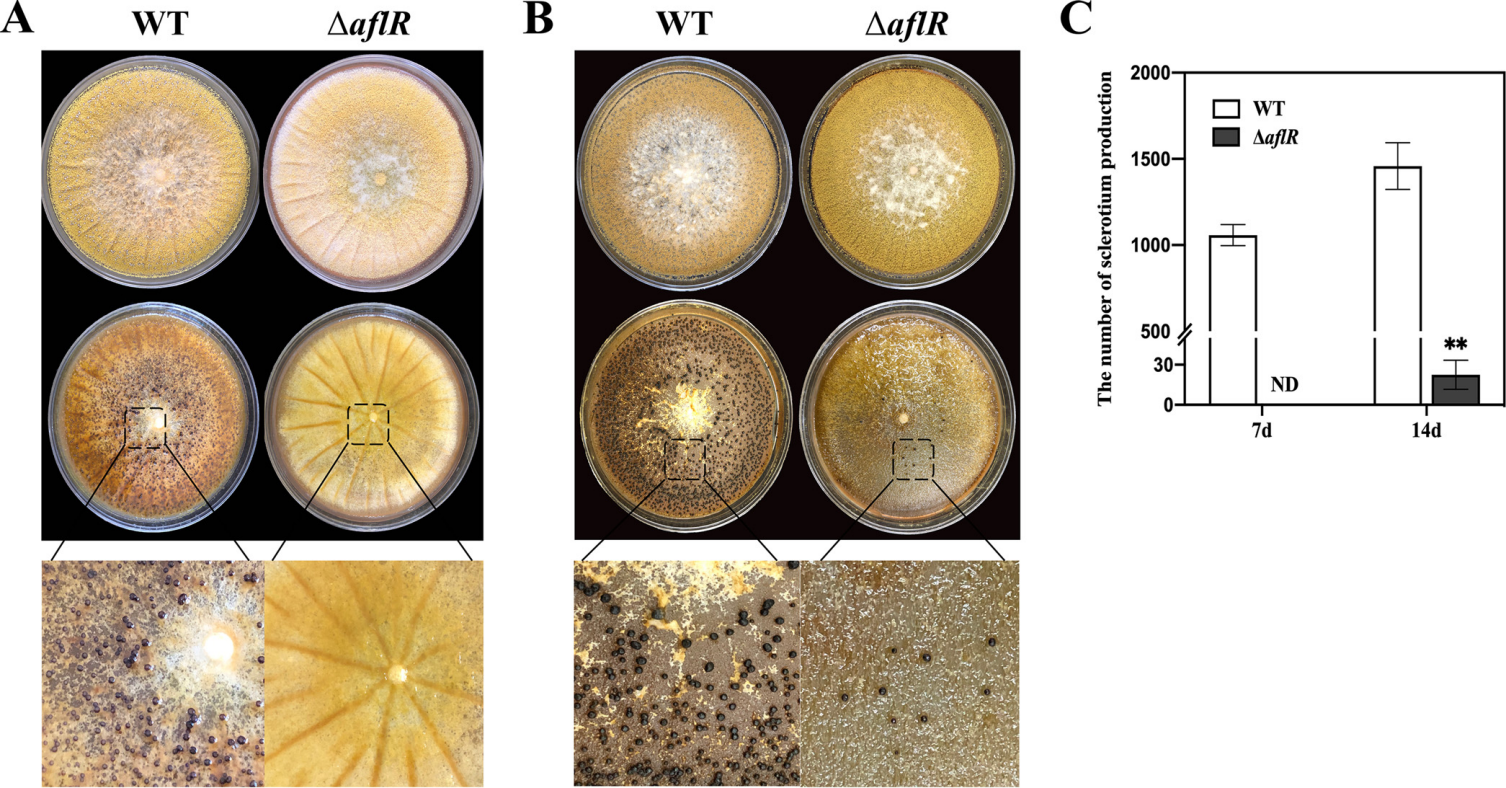
Sclerotial development and production of the WT and Δ*aflR* strains. (A) Top panels: colony morphology of the WT strain (left side) and the Δ*aflR* strain (right side) grown on WKM medium at 30°C for a week in the dark. Bottom panels: closeup of the central area that shows sclerotia produced by the Δ*aflR* strain in comparison to that of the WT strain. (B) Sclerotial development of the WT and Δ*aflR* strains after 14 days. (C) Sclerotial production of the WT and Δ*aflR* strains at day 7 and day 14.

The Wickerham medium was used to research sclerotial development in the WT, Δ*aflR*, and OE strains. The growth of sclerotia of the WT and Δ*aflR* strains was observed after 7 days and 14 days. The WT strain produced many immature sclerotia on the seventh day and that appeared brown in color, while the Δ*aflR* strain had no sclerotia on the seventh day (Fig. 4A). After 14 days, the WT and OE strains produced a large number of mature sclerotia, while the Δ*aflR* strain produced only a much smaller number of sclerotia. On the seventh day the sclerotia produced by the WT strain exceeded 1,000 on each plate. On the 14th day, the average yield of the Δ*aflR* strain sclerotia was 22.5 per plate, and the average yield of the WT strain sclerotia reached 1,458 per plate, which was about 65 times that of the Δ*aflR* strain (Fig. 4C). The average yield of sclerotia of the OE strain was about 3,733 per plate, which was significantly higher than that of the WT strain. (Fig. S6).

### Effect of the *aflR* deletion on aflatoxin and CPA biosynthesis.

**FIG 5:**
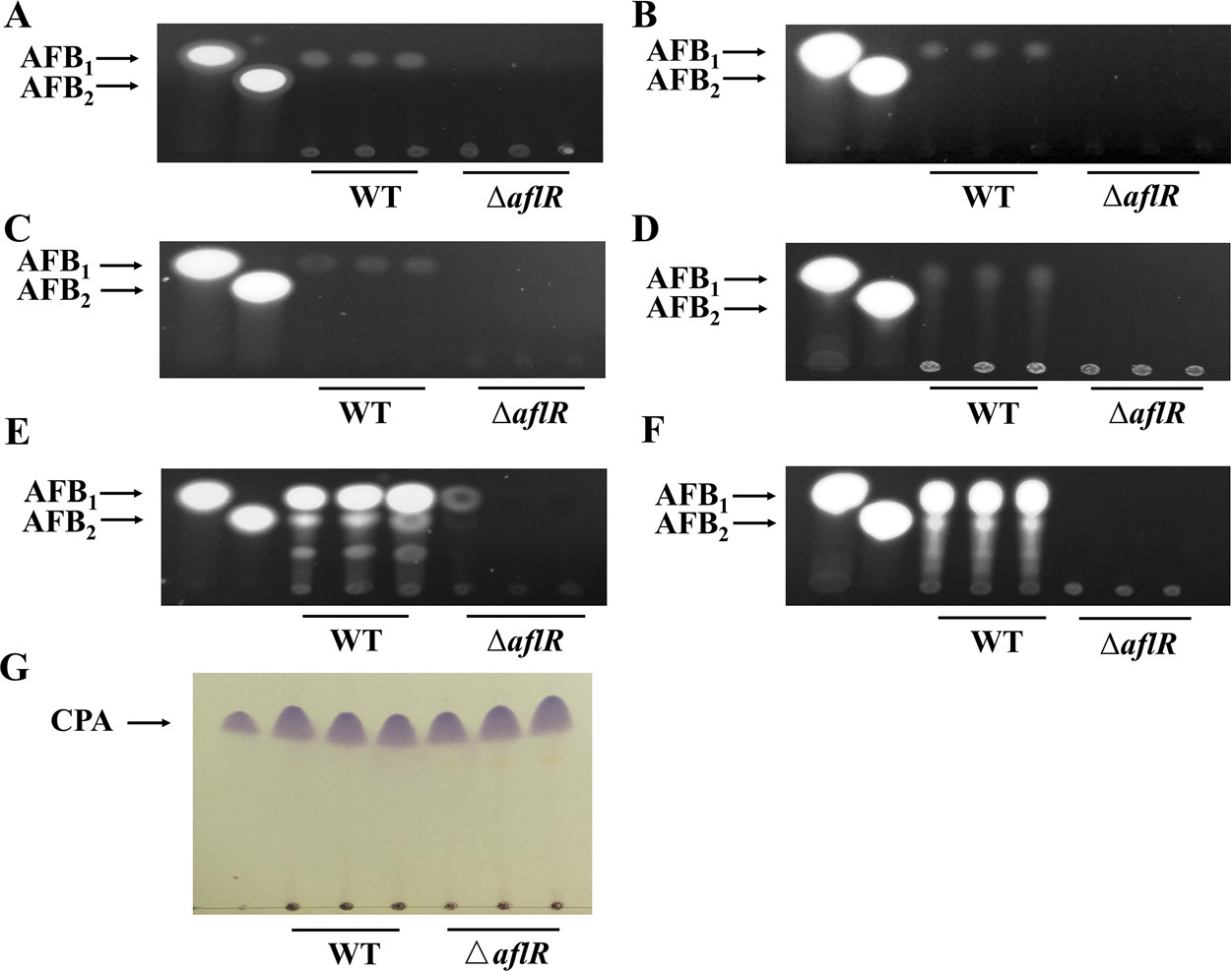
AFB production of the WT and Δ*aflR* strains after 5 days (A, C, E) and 7 days (B, D, F) and CPA production of the WT and Δ*aflR* strains after 7 days (G). AFB yields on GMM (A, B), PDA (C, D), and YES (E, F) were determined by TLC.

Production of AFB_1_ and AFB_2_ by the WT and Δ*aflR* strains were determined using thin-layer chromatography (TLC) after 5 and 7 days of growth on different media (Fig. 5), and the highest yield of the WT and Δ*aflR* strains was obtained on YES medium. The AFB_1_ and AFB_2_ production of the WT and Δ*aflR* strains cultured on YES medium for 5 days was also determined using HPLC. The AFB_1_ of Δ*aflR* strain was 180 ng/ml and the AFB_2_ was 2.95 ng/mL (Table 1). The AFB_1_ and AFB_2_ content of OE strains were determined after 7 days of growth on YES medium. TLC showed that the AFB_1_ and AFB_2_ content of OE strain were higher than that of the WT strain (Fig. S7). The production of CPA was determined using TLC for the WT and Δ*aflR* strains (Fig. 5G) where CPA was colored as purple spots by the chromogenic agent. There was no significant difference in CPA production between the Δ*aflR* and WT strains.

**TABLE 1:** AFB produced by the WT and Δ*aflR* strains

Aflatoxins	WT	Δ*aflR*
AFB_1_	136 μg/mL	180 ng/mL^*a*^
AFB_2_	2.43 μg/mL	2.95 ng/mL^*a*^

a Data results were the average of three parallel experiments. Error range of all values are between ± 3% −8%. The actual error values were omitted in the table.

### Transcriptome analysis of WT and Δ*aflR* strains.

**FIG 6:**
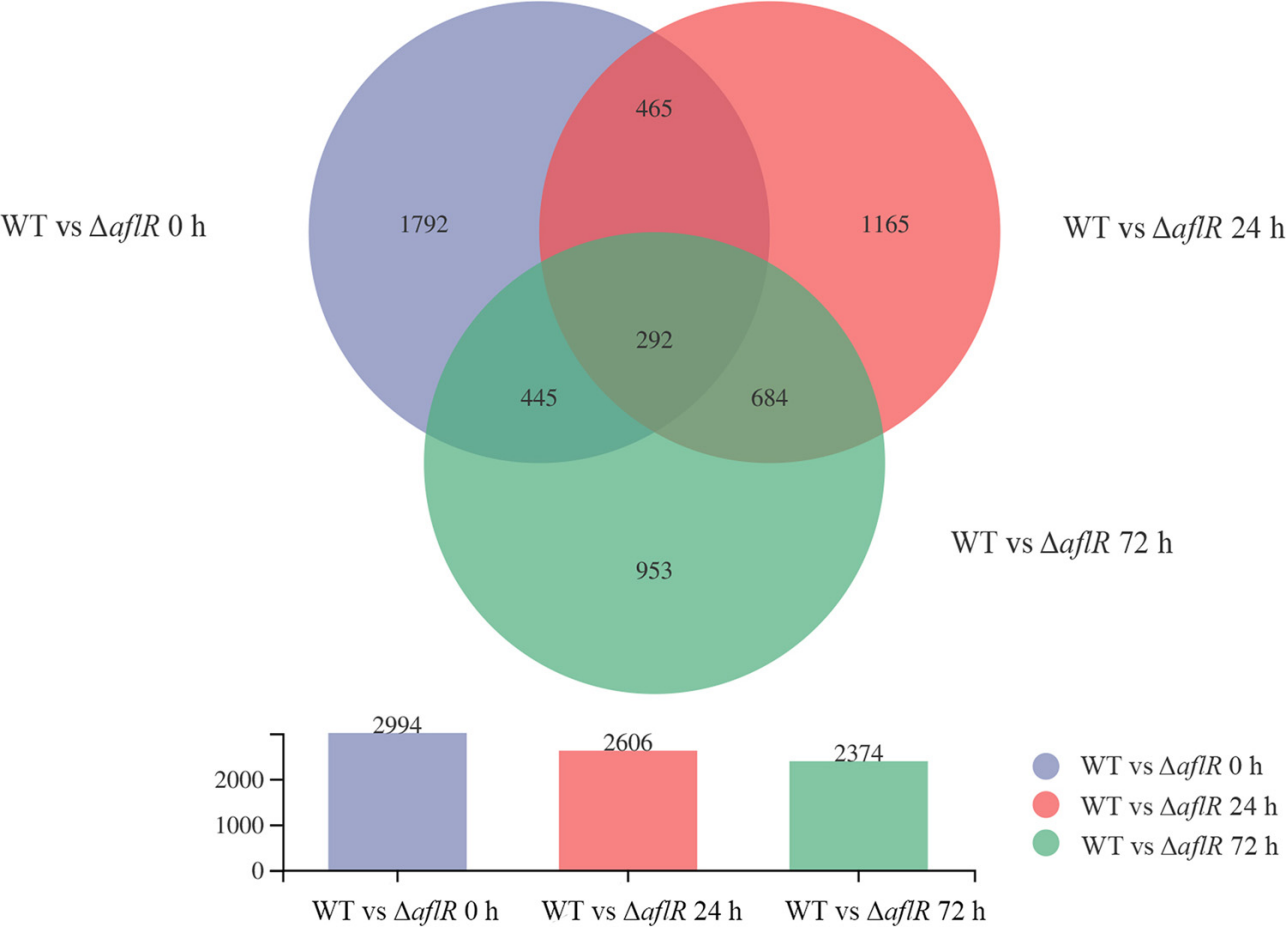
Venn analysis for the WT and Δ*aflR* strains.

In this study, 2,994 differentially expressed genes (DEGs) were identified at 0 h, of which 1,606 were downregulated DEGs and 1,388 were upregulated DEGs (Data set S1). At 24 h, 2,606 DEGs were identified, of which 1,268 were downregulated and 1,338 were upregulated (Data set S2). At 72 h, 2,374 DEGs were identified, of which 1,201 were downregulated and 1,173 were upregulated (Data set S3). The WT and Δ*aflR* strains shared 757 common DEGs at 0 h and 24 h, 737 common DEGs at 0 h and 72 h, 976 common DEGs at 24 h and 72 h, and 292 common DEGs at 0 h, 24 h, and 72 h (Fig. 6).

**FIG 7:**
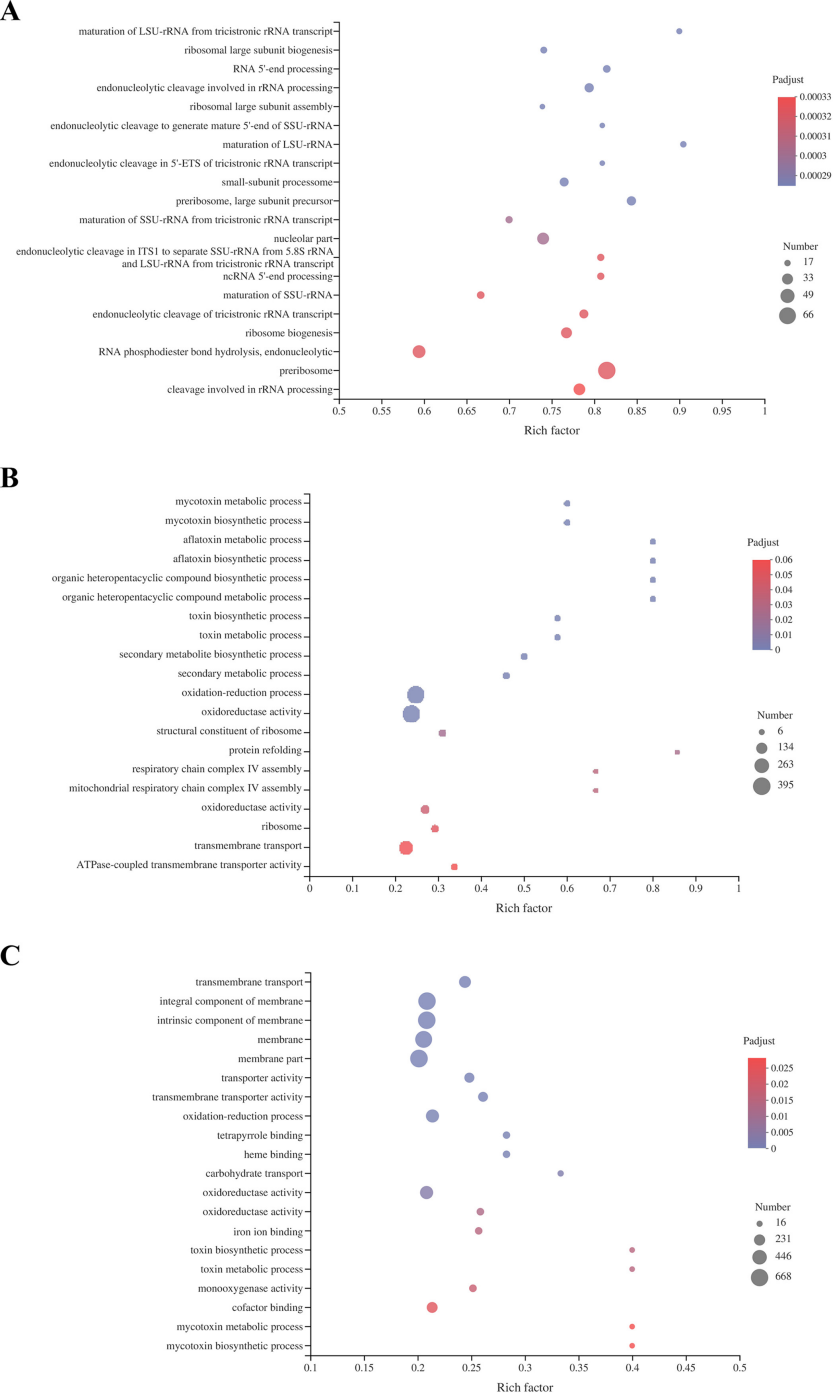
GO enrichment analysis of the WT and Δ*aflR* strains (A, 0 h; B, 24 h; C, 72 h). The rich factor represents the ratio of the number of DEGs annotated as GO functional classes to the number of all identified genes annotated as GO functional classes.

DEGs identified at 0 h, 24 h, and 72 h were subjected to Gene ontology (GO) enrichment analysis (Fig. 7A). Enriched GO terms for DEGs of the Δ*aflR* strain at 0 h included maturation of LSU-rRNA from tricistronic rRNA transcript, ribosomal large subunit biogenesis, and RNA 5′-end processing. At 24 h, enriched GO terms for DEGs of the Δ*aflR* strain included mycotoxin metabolic process, mycotoxin biosynthetic process, aflatoxin metabolic process, and aflatoxin biosynthetic process (Fig. 7B). At 72 h, the GO function enrichment of DEGs for the Δ*aflR* strain showed significant changes including transmembrane transport, membrane components, transport activity, toxin biosynthesis process, toxin metabolism process, mycotoxin metabolism process, and mycotoxin biosynthesis process, etc. (Fig. 7C). At 24 h and 72 h, GO terms for the WT and Δ*aflR* strains were mainly enriched in processes related to toxin biosynthesis and toxin metabolism, including aflatoxin biosynthesis and metabolism.

**FIG 8:**
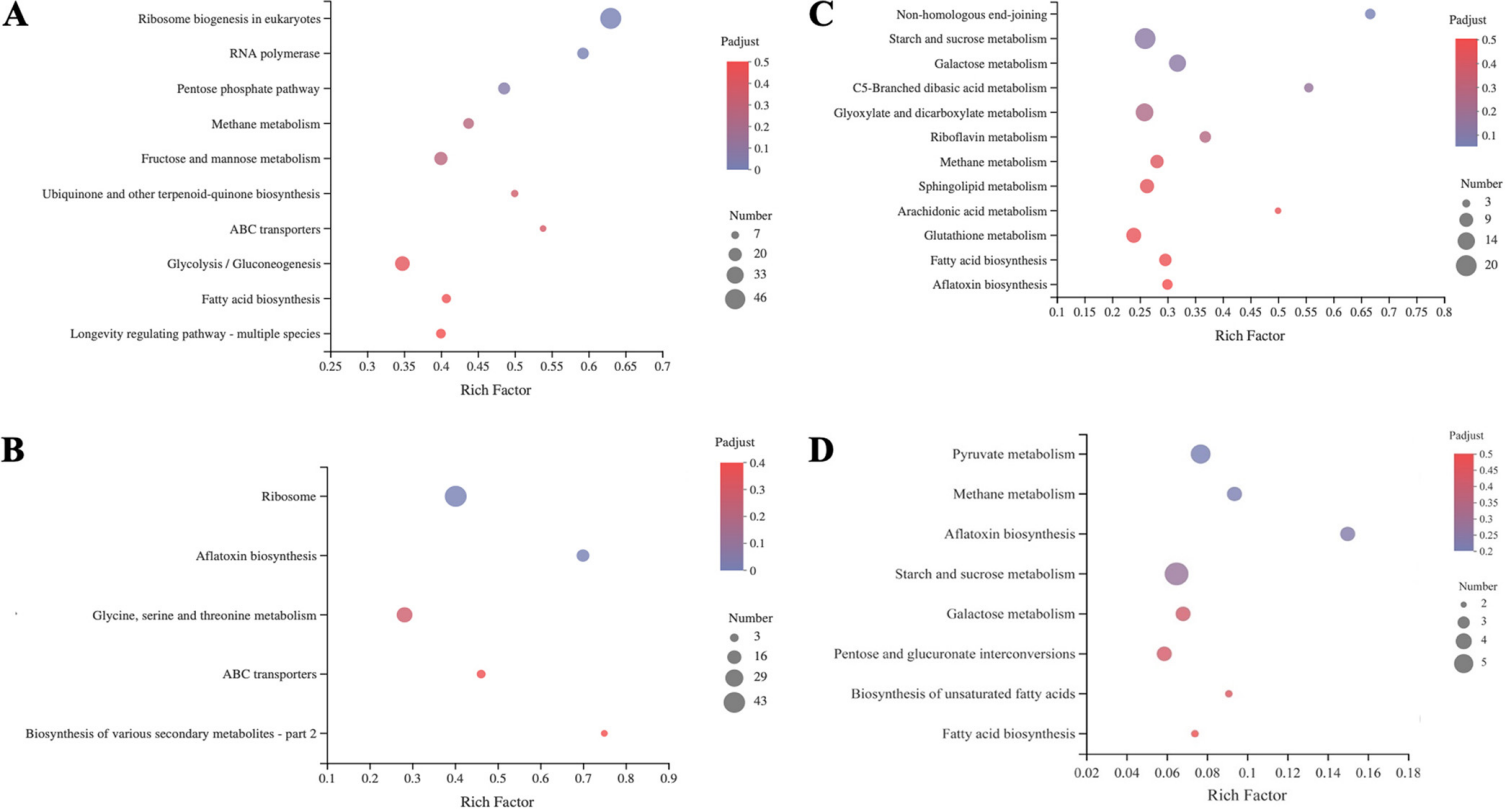
KEGG enrichment analysis of the WT and *ΔaflR* strains (A, 0 h; B, 24 h; C, 72 h) and KEGG enrichment analysis of common DEGs of the WT and *ΔaflR* strains at 0 h, 24 h, and 72 h (D). The rich factor indicates the ratio of the number of DEGs involved in the KEGG pathway to the number of genes involved in the pathway among all identified genes.

KEGG pathway enrichment analysis further performed on DEGs of the WT and Δ*aflR* strains. The significantly different metabolic pathways of the Δ*aflR* strain compared with the WT strain at 0 h, 24 h, and 72 h (Fig. 8). Secondary metabolism especially aflatoxin biosynthesis was significantly affected by the *aflR* deletion at 24 h, especially aflatoxin biosynthesis. Additionally, the common 292 DEGs obtained by Veen analysis at 0 h, 24 h, and 72 h were analyzed to KEGG pathway enrichment analysis to find metabolic pathways significantly associated with *aflR*. The pathways identified were those involved in pyruvate metabolism, methane metabolism, aflatoxin biosynthesis, starch and sucrose metabolism, galactose metabolism, interconversions of pentose and glucuronate interconversions, biosynthesis of unsaturated fatty acids, and fatty acid biosynthesis pathways (Fig. 8D).

## DISCUSSION

### Impacts of *aflR* on mycelial growth and conidial formation.

**FIG 9:**
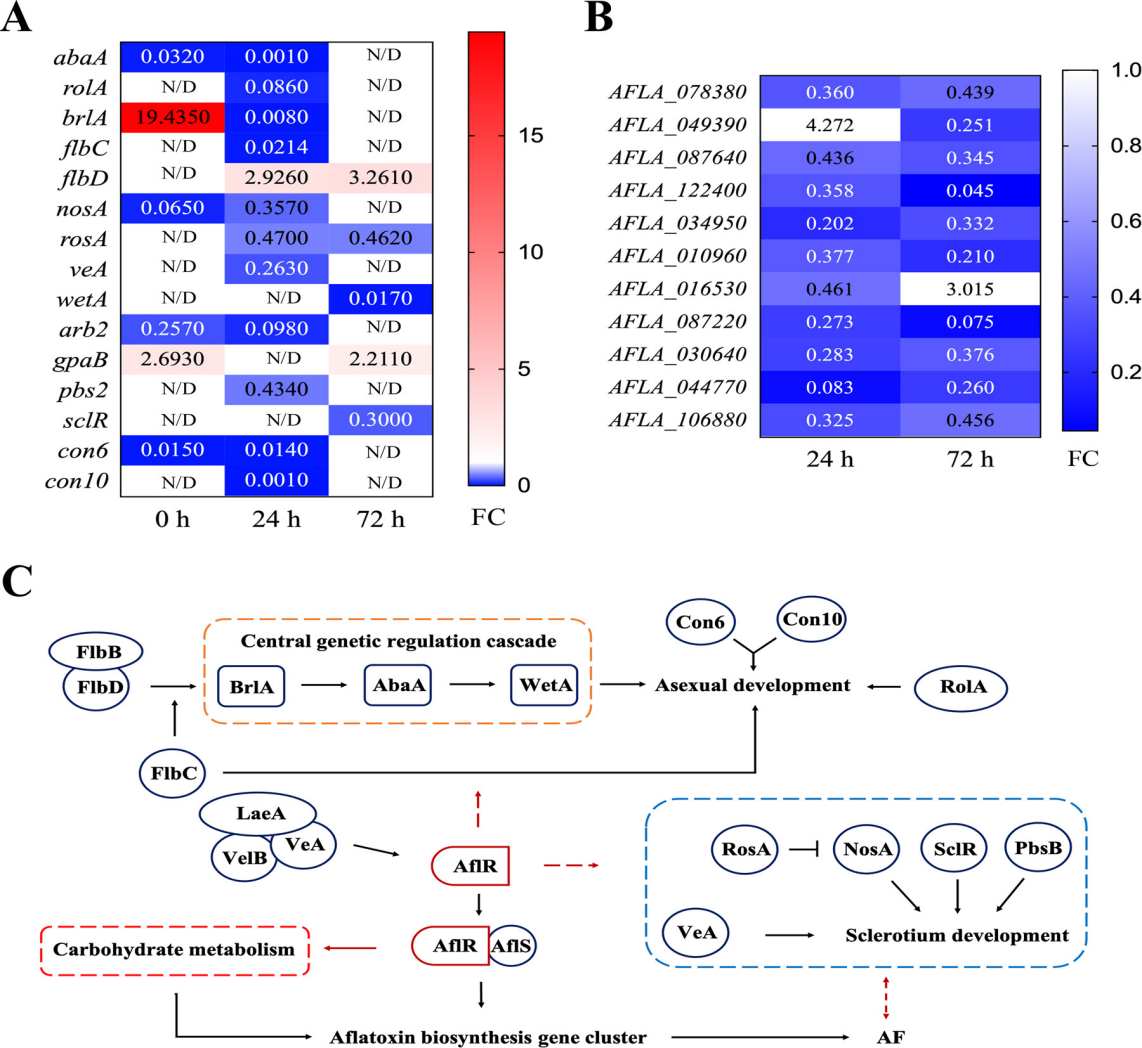
Effect of *aflR* knockout on the expression of growth and development-related genes (A) and carbohydrate metabolism (B) of A. flavus compared with the WT strain, and the putative model of the comprehensive regulation of A. flavus (C).

Growth and development are the two phases of asexual reproduction that Aspergillus undergoes. The growth period includes conidial germination and mycelial formation. When nutrition is limited, mycelial cells stop growth by forming complex structures (becoming conidia) and begin asexual reproduction (23). In the present study, we found significant differences in the expression of many genes related to the growth and development of A. flavus in the Δ*aflR* strain (Fig. 9A) and their associated functions are summarized as follows (Table 2). The *abaA* gene plays a crucial role in the differentiation of phialides, the hyphal cells necessary for the formation of conidia (24). The *abaA* gene also affects secondary metabolism by regulating the expression of *veA*, *velB*, and *velC* (25). The *brlA* gene encodes a C_2_H_2_ zinc finger transcription factor (TF), a key regulator of Aspergillus conidia production, which mediates vesicle formation and budding cell growth during the early stages of asexual development (26, 27). The *wetA* gene plays a key role in the coordinated control of Aspergillus spore production, though the exact molecular mechanism of *wetA* is not known, deletion of *wetA* results in defects in conidiophore development (23). The *wetA* gene activated by *abaA* completes the developmental role in the late stage of conidiation (28). The wetA gene at 72 h in the ΔaflR strain was only 0.017-fold of the WT strain. The significant decreased expression of the *wet* gene in the Δ*aflR* strain, likely caused the related defects in conidial formation. The central genetic regulatory cascade formed by *brlA*→*abaA*→*wetA* acts in concert with other genes to control gene expression in spore-specific cells and to determine the sequence of gene activation in spores during cellular and chemical development (28).

**TABLE 2:** Growth and development related genes and their functions affected by *aflR* gene knockout

Gene	Associated function
*abaA*	Transcription factor for conidia formation
*rolA*	Encoding hydrophobic protein
*brlA*	C_2_H_2_ zinc finger protein transcriptional activator of conidiophore
*flbC*	Putative C_2_H_2_ conidiation transcription factor
*flbD*	MYB family conidiophore development
*nosA*	Necessary for the induction of gene transcription in filamentous fungi in sexual reproduction
*rosA*	Zn2-Cys6 binuclear cluster domain-containing protein
*veA*	Global regulator
*wetA*	Developmental regulatory protein
*arb2*	Conidial pigment biosynthesis oxidase
*gpaB*	Asexual sporulation, AF biosynthesis, and virulence by regulating cAMP signaling
*pbs2*	Involved in the growth of mycelium, conidiation, and sclerotia formation
*sclR*	Involved in mycelial morphology, conidial development, and sclerotial formation
*con6*	Conidiation protein
*con10*	Conidiation-specific protein

The *flbC* and *flbD* genes regulate the expression of the *brlA* gene upstream to control the asexual development of Aspergillus (29). Disruption of the *flbC* gene resulted in delayed spore germination and reduced expression of the *brlA* gene, while FlbC bound to *abaA* and *vosA* play a regulatory role (30). FlbD is a c-Myb transcription factor, and deletion of the *flbD* gene results in a fluffy phenotype. Overexpression of *flbD* leads to sporulation in a liquid culture environment, which is caused by a failure of *brlA* activation (31). The FlbB/FlbE complex is required for the expression of *flbD*, and mechanisms exist for both FlbB/FlbE and FlbD to activate *brlA* gene expression (32). However, the activation of the *brlA* gene seems to be the result of the accumulation of multiple pathways (30). In this study, the expression of *flbC* in the Δ*aflR* strain was significantly reduced at 24 h, which was only 0.021 times that of the WT strain, while the expression of *flbD* was significantly upregulated at 24 h and 72 h to 2.926 and 3.261 times that of the WT strain, respectively. This may be due to the presence of multiple pathways activating *brlA* gene expression, and when one of the pathways is repressed, the repressive effect is offset by an increase in expression of another pathway. However, in the Δ*aflR* strain, the extent of *flbC* downregulation fold exceeded that of *flbD*, which is one of the possible reasons why *brlA* was not effectively activated.

Suzuki et al. found that a single deletion of *con* genes in A. nidulans did not cause significant phenotypic changes, while simultaneous inactivation of two or three *con* genes would result in delayed spore germination (33). Among them, *con6* and *con10* were representatives of *con* genes preferentially expressed during conidiation (34, 35). In this study, *con6* and *con10* were significantly downregulated in Δ*aflR* strain, which seems to be the reason for the limited spore formation in the Δ*aflR* strain compared with the WT strain.

Hydrophobin proteins are effective regulators that play a structural or enzymatic role in the formation of asexual developmental structures (36). The A. flavus hydrophobin protein is encoded by the *rolA* (*rodA*) gene, and deletion of the *rolA* gene results in reduced hydrophobicity of conidia (37). The expression of *rolA* gene may affect aflatoxin biosynthesis. For example, the inhibition of aflatoxin biosynthesis by Bacillus megaterium was related to significant inhibition of *rolA* gene expression (38). The results showed that the *aflR* gene could affect conidial development, but the *aflR* gene deletion did not cause complete impairment of conidial development, indicating that the *aflR* gene was not a key gene for the growth and development of A. flavus colonies and spores.

### Regulation of sclerotial formation by *aflR*.

Sclerotium is another way of Aspergillus reproduction. Aspergillus hypha branches aggregate into a dense network to form the initial white immature sclerotia, and then the immature sclerotia with pigment deposition form mature sclerotia (8, 39). Mature mycorrhizal sclerotia contain a large number of metabolites such as aflatoxins and indoles. The sclerotium, as an important asexual reproduction structure for the spread of A. flavus in the soil and its growth in adverse conditions, is related to secondary metabolites. The gene *nosA* is one of the genes necessary for the induction of gene transcription in filamentous fungi in sexual reproduction (40). In A. fumigatus, *nosA* knockout led to impaired sexual reproduction in the fungus, and *nosA* expression was found to correlate with virulence (40). Inactivation of RosA did not affect asexual and sexual reproduction when VeA was present, indicating that the function of the *rosA* gene in asexual and sexual reproduction depends on *veA* level (41). In this study, both *nosA* and *rosA* were significantly downregulated in the Δ*aflR* strain. However, *rosA* is a developmental repressor gene whose downregulation should be positively regulating sexual reproduction. This may be because the *rosA* gene functions under the influence of *veA*, and significant downregulation of the *veA* gene prevents *rosA* from functioning properly. In A. oryzae, the *sclR* gene plays an important role in mycelial morphology, conidial development, and sclerotial formation, and overexpression of *sclR* promotes high mycelial aggregation to initiate sclerotia formation (42). The *sclR* gene was significantly downregulated in the Δ*aflR* strain at 72 h, which was only 0.300-fold of the WT strain. The *veA* gene functions as a global regulator of a variety of morphogenetic and secondary metabolic genes in fungi and is one of the genes essential for fungal growth and development (43). In the nucleus, three proteins, VeA, LaeA, and VelB, form a heterotrimeric velvet complex to coordinate and control the development and secondary metabolism of the fungi (25). In this study, a significant downregulation of *veA* at 24 h was observed in the Δ*aflR* strain, which was only 0.263 times that of the WT strain. In this study, *veA* of the Δ*aflR* strain was significantly downregulated to 0.263 times that of the WT strain at 24 h, which may affect its morphogenesis and secondary metabolism.

### Effect of *aflR* on metabolic pathways.

The mitogen-activated protein (MAP) kinase cascade reaction is one of the mechanisms by which eukaryotic cells transfer information from the extracellular environment to target gene expression in the nucleus via plasma membrane-associated receptors, and the MAP kinase signaling pathway regulates development and toxin biosynthesis (44). The *pbs2* gene was significantly downregulated in the Δ*aflR* strain at 24 h. *pbs2* (*pbsB*), as MAPKK, is critical in the growth and virulence of A. flavus (45).

Carbohydrate metabolism plays a key role in fungal secondary metabolism and fungal infection, and fungal adaptation to changes in the extracellular environment; it is critical to their growth and development under stress conditions (46, 47). Pyruvate metabolism, starch, and sucrose metabolism, and the interconversion of pentose and glucuronide are parts of carbohydrate metabolism, and genes associated with these metabolic pathways were significantly downregulated in the Δ*aflR* strain. Therefore, *aflR* apparently affects carbohydrate metabolism. Kong et al. found that AfAflR can bind to genes encoding carbohydrate transporters, which suggests that *aflR* is involved in carbohydrate metabolism and there may regulate carbohydrate metabolism (19). Carbohydrate-related metabolic pathways were significantly altered after *aflR* knockdown, consistent with the findings of Kong et al. (Fig. 9B).

To sum up, Fig. 9C shows a presumptive model map on the full regulation of growth and development of A. flavus. AflR may regulate the process of conidial germination by regulating the central genetic regulatory cascade *brlA*→*abaA*→*wetA* genes and other genes related to conidial development, such as *flbD*, *flbC*, *rolA*, *con6*, and *con10*. It also may regulate the formation and development of sclerotia by regulating the expression of *rosA*, *nosA*, *pbsB*, and *sclR*.

### *aflR* gene regulates gene expression of aflatoxin biosynthesis gene cluster.

**FIG 10:**
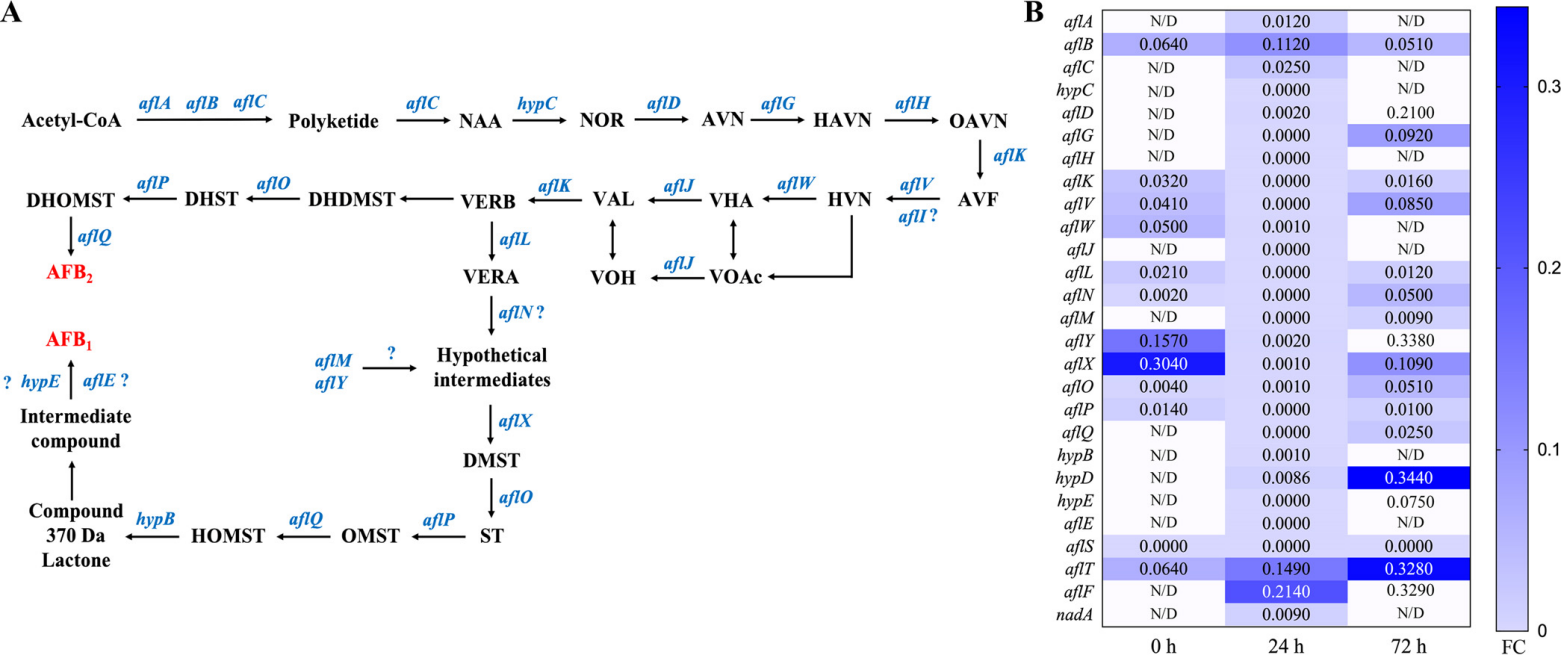
Effect of *aflR* knockdown on the aflatoxin biosynthesis pathway (A) and on the expression profile of genes in the pathway (B). FC, fold change; N/D, not detected.

The aflatoxin biosynthetic pathway has long been regarded as one of the most complex metabolic pathways of natural secondary metabolites, involving at least 27 enzymatic reactions (11, 48). In this study, the transcriptomic analysis revealed that genes of the aflatoxin biosynthesis gene cluster were significantly downregulated (Fig. 10). At 0 h, most aflatoxin biosynthesis genes were not expressed by both WT and Δ*aflR* strains. In particular, all 27 genes on the aflatoxin biosynthetic pathway were significantly downregulated at 24 h (*P < *0.01). A total of 13 genes, *hypC*, *aflG*, *aflH*, *aflK*, *aflV*, *aflJ*, *aflN*, *aflM*, *aflP*, *aflQ*, *hypE*, *aflE*, and *aflS*, were not expressed in the Δ*aflR* strain at 24 h. In contrast, at 24 h, all genes on the aflatoxin biosynthetic gene cluster of the A. flavus were normally expressed. The aflatoxin biosynthetic pathway of the Δ*aflR* strains is disrupted, indicating that the *aflR* gene, as an important transcriptional regulator in the aflatoxin biosynthetic gene cluster, is one of the indispensable genes in the aflatoxin biosynthetic pathway. AflR binding sites were present in at least 17 genes in the aflatoxin biosynthesis pathway (13). Interestingly, the *aflS* gene was not expressed after *aflR* knockdown at 0 h, 24 h, or 72 h. In addition, the knockout of the *aflR* gene resulted in a small amount of aflatoxin production. Although genes related to the aflatoxin biosynthetic pathway were significantly downregulated, these genes were still expressed at low levels. These likely resulted from basal expression of those aflatoxin pathway structural genes after the *aflR* gene was deleted, resulting in the eventual possibility of still producing low levels of aflatoxin. Combined phenotypic and transcriptomic data analysis showed that the *aflR* gene not only affected the aflatoxin biosynthesis but also played a positive role in asexual reproduction, sclerotial development, and growth of A. flavus.

## MATERIALS AND METHODS

### Strains and culture conditions.

Fungal strains used in this study included A. flavus NRRL3357 (WT strain), TJES19.1 (Δ*pyrG*, Δ*Ku70*) (49), Δ*aflR* strain (Δ*aflR*, Δ*Ku70*, *pyrG*^+^), and OE strain (*gpdA^+^*, *aflR^+^*,Δ*Ku70*, *pyrG*^+^). All strains were cultured at 30°C and stored short-term at −20°C (long-term, −80°C) in a glycerol stock solution. WT, Δ*aflR*, and OE strains stored at −20°C were spotted with 3 μL on V8 juice media for activation and incubated at 30°C for 5 days. The spores at 10^8^ spores/mL were harvested from V8 juice medium (5% V8 juice, 2% agar, pH 5.2) using 0.1% Triton X-100 solution and inoculated in 100 mL Czepak-Dox Broth (supplemented with 0.5% casein amino acids, 10 mM ammonium sulfate, 0.2% uracil and 1% glucose) after resuspension with sterilized water.

### Construction of strains.

Gene replacement by double crossover recombination was carried out in strains lacking the non-homologous end joining (NHEJ) (50). The Δ*aflR* strain were constructed by a deletion cassette. The two flanking fragments of the *aflR* gene and the screening marker gene *pyrG* were amplified using PrimeSTAR HS DNA polymerase (TaKaRa Bio Co., Ltd., Japan). The deletion cassette was generated by the fusion PCR (Fig. S1A) (51). The *aflR* gene was overexpressed via Gibson Assembly Cloning Kit (New England Bio Co., Ltd., USA). 5′-UTR, *pyrG*, *gpdA*, *aflR*, and puC19 were amplified and overlapped with particular primers. A molar ratio of 1:1 between vector and each fragment should be used. In a final volume of 20 μL at 50°C, 10 μL of 2X Gibson Assembly Master Mix was incubated with five fragments (0.05 pmol each) for 60 min. All primers used in the study were shown in Table S1.

TJES19.1 was transformed as described by Chang et al. (50). The obtained transformants were cultured in a regeneration medium (Czepak-Dox Broth supplemented with 0.6 M KCl, 5 mM ammonium sulfate) for 7 to 10 days and then validated by PCR and sequencing (Fig. S1B, Fig. S2). There is an intergenic region (491 bp) between *aflR* and *aflS*, and the primers FAflS and RAflS were used to amplify and check the intact *aflS* gene band in the *aflR* knockout positive transformants, and the selected PCR-positive transformants were purified (E.Z.N.A Cycle Pure Kit D6492, Omega Bio-tek Ltd., USA) and verified by sequencing (commissioned by RuiBiotech, Beijing, China) (Fig. S3).

### Mycelial growth and conidial formation.

Approximately 3 μL 10^6^ spores/mL of WT and Δ*aflR* strains were spotted in the center of GMM (52), PDA (Beijing Solarbio Science & Technology Co., Ltd., Beijing, China), and YES media and incubated at 30°C for 5 days. After 3 days in GMM medium, a small piece of fungal culture was removed, placed on a microscope slide, and a drop of 0.85% NaCl solution was added to the fungal culture for microscopic observation. The morphology of conidial were also recorded at 24 h, 30 h, 36 h, 42 h, and 48 h.

Conidia were determined as follows. A spore suspension of approximately 3 μL 10^8^ spores/mL was spotted in the center of GMM, PDA, and YES media and incubated at 30°C for 3, 5, and 7 days. Agar plugs of 1.5 cm in diameter media (three samples per plate, three replicates per strain) were sampled and transferred to a 10-mL centrifuge tube, 5 mL of 0.1% Tween 80 solution was added and vortexed for 5 min. Then the agar plugs were removed, and spores were counted using a hemocytometer.

### Sclerotial formation analysis.

Sclerotial formation analysis was performed as follows. Approximately 3 μL 10^8^/mL WT and Δ*aflR* spore suspensions were inoculated in the center of the Wickerham medium (8). After grown at 30°C for 7 and 14 days, fungal cultures were sprayed with 95% ethanol, and the sclerotia were exposed and counted.

### Determination of aflatoxin and semi-quantitative determination of cyclopiazonic acid.

The TLC analytical method for aflatoxins was modified (53). Strains were incubated on GMM, PDA, and YES for 5 and 7 days at 30°C. Three agar plugs (1.5-cm diameter), 1 cm from each inoculation site, were cored with transfer tubes and placed in 10 mL centrifuge tubes and extracted twice, each time with 5 mL of methanol (three replicates per strain). The extracts were vortexed for 3 to 5 min at 30°C and shaken at 200 rpm for 45 min. The supernatant was transferred, centrifuged at 4,000 g for 15 min, blown dry with nitrogen at 55°C, and resuspended with 1 mL CHCl_3_. Then, 10 microliters (2.5 μL/time) of WT and Δ*aflR* aflatoxin extracts were spotted onto a TLC plate. The plate (105554, Merck, Germany) was developed in methanol:ethyl acetate:acetic acid (96:3:1 vol/vol) and air-dried. Aflatoxin was visible under 365 nm UV light. High-performance liquid chromatography (HPLC) determination of aflatoxin produced by 5-day-old cultures on YES at 30°C was entrusted to Pony Testing International Group (Beijing, China). HPLC method for AFB_1_ and AFB_2_ was referenced to national standards of P. R. China (GB 5009.22-2016).

To determine the CPA production of the WT and Δ*aflR* mutants, approximately 3 μL of 10^8^ spores/mL spore suspension was spotted in the center of each Wickerham medium plate and incubated at 30°C for 7 days. Agar plugs of 1.5 cm in diameter media (three samples per plate, three replicates per strain) were sampled and transferred to a 5-mL centrifuge tube; 3 mL CHCl_3_ was added, vortexed for 5 min, and shaken at 30°C for 45 min. The supernatant was removed and determined by TLC following the method of Chang et al. (54).

### Transcriptome analysis of the WT and Δ*aflR* strains.

**(i) Preparation of transcriptome samples and RNA extraction.** Approximately 10^8^ spores/mL of the WT and Δ*aflR* strains were inoculated in the PDB medium and grown for 24 h at 30°C 180 rpm. Mycelia were collected through sterilized Miracloth and washed with sterile water. The water on the surface of the mycelia was blotted out. The mycelia were spread flat on PDA plates at 30°C and sampled at 0 h, 24 h, and 72 h. The removed samples were quickly snap-frozen in liquid nitrogen for 1 h and stored at −80°C. Total RNA was extracted using the TRIzolTM kit (Thermo Fisher Scientific, USA) and DNA was removed using DNase I (TaKaRa, Japan). RNA integrity was examined using RNA electrophoresis and RNA quality was determined using an Agilent 2100 Nano (Agilent Technologies, Palo Alto, CA, United States). The concentration of RNA was determined using NanoDrop 2000 (Agilent Technologies, Palo Alto, CA, USA).

**(ii) RNA-seq and enrichment analysis of differentially expressed genes**. The total RNA of three biological replicates for the Δ*aflR* and WT strains were sequenced. RNA libraries were generated using the TruSeq^TM^ RNA sample preparation kit (Illumina, USA). Raw sequencing data were converted to sequence data (clean data) by base calling and stored in fastq format. The clean data were blasted to the reference genome of the A. flavus genome sequence, and the mapped data (reads) were obtained for subsequent analysis. Cufflinks counts mapped clean reads for each gene and normalizes them to fragment per thousand base pairs of transcribed fragments mapped reads per million (FPKM) values (55). Expression profiles in genes and transcripts were quantified by Salmon expression quantification software (56). A gene was treated as significantly DEGs when |log_2_(fold change)| ≥ 2 with an adjusted *P value* ≤ 0.05 (Data set S1, Data set S2, Data set S3). GO terms (including cellular component, molecular function, and biological process) and Kyoto Encyclopedia of Genes and Genomes (KEGG) pathways were classified as significantly enriched among differentially expressed genes (*P < *0.05).

### Statistical analysis.

The data were processed by SPSS 25 software and significance analysis (*P < *0.05) was performed.

### Data availability.

The results of the RNA-seq data were submitted to NCBI's GEO database and assigned accession no. GSE179978.
