# Supplementary material for: New Insights of Transcriptional Regulator AflR in Aspergillus flavus Physiology
Source: Microbiol Spectr. 2022 Jan 26;10(1):e00791-21. doi: 10.1128/spectrum.00791-21 (PMC8791188; doi:10.1128/spectrum.00791-21)
Supplement: SUPPLEMENTAL FILE 4 — Supplemental material. Download SPECTRUM00791-21_Supp_4_seq14.pdf, PDF file, 0.7 MB [file spectrum00791-21_supp_4_seq14.pdf]

New insights of transcriptional regulator AflR in *Aspergillus flavus* physiology

Peng Wang[1#], Jia Xu[1#], Perng-Kuang Chang[2], Zhemin Liu[1], Qing Kong[1*]

[1] School of Food Science and Engineering, Ocean University of China, Qingdao, Shandong 266003, China

[2] Southern Regional Research Center, Agricultural Research Service, US Department of Agriculture, New Orleans, Louisiana 70124, United States

[#] These authors contributed to this work equally.

***Address for correspondence:** Qing Kong, School of Food Science and Engineering, Ocean University of China, Qingdao. Yushan Road 5, 62 Building, Qingdao, Shandong 266003, China

E-mail: kongqing@ouc.edu.cn

Tel: +86-532-8203-2290

Fax: +86-532-8203-2389

Supplementary materials

Fig. S1 Diagram of transformant identification by PCR (A) and electrophoresis diagram of transformant identification by PCR (B).

Fig. S2 Comparison of knockout cassette and Δ*aflR* strain recombinant region sequencing.

Fig. S3 Comparison of WT and Δ*aflR* strain *aflS* gene sequencing.

Fig. S4 Different morphology of conidial of the WT and *ΔaflR* strains at 24 h, 30 h, 36 h, 42 h, and 48 h.

Fig. S5 Conidial production of the WT and OE strains on different media at day 3 (A), day 5 (B), and day 7 (C).

Fig. S6 Sclerotial development and production of the WT and OE strains. (A) Sclerotial development of the WT and OE strains at day 14. (B) Sclerotial production of the WT and OE strains at day 14.

Fig. S7 AFB production of the WT and OE strains after 7 days.

Table S1 All primers used in the study

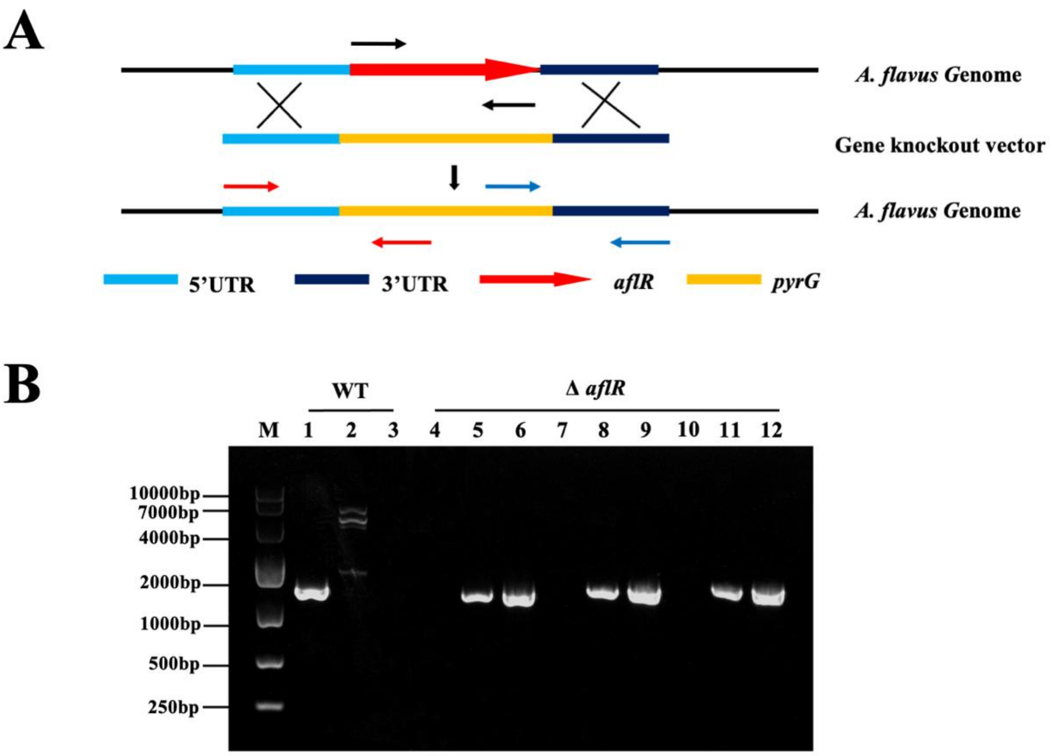
A
A. flavus Genome
Gene knockout vector
A. flavus Genome
5'UTR
3'UTR
aflR
pyrG
B
WT
Δ aflR
M 1 2 3 4 5 6 7 8 9 10 11 12
10000bp
7000bp
4000bp
2000bp
1000bp
500bp
250bp

Fig. S1

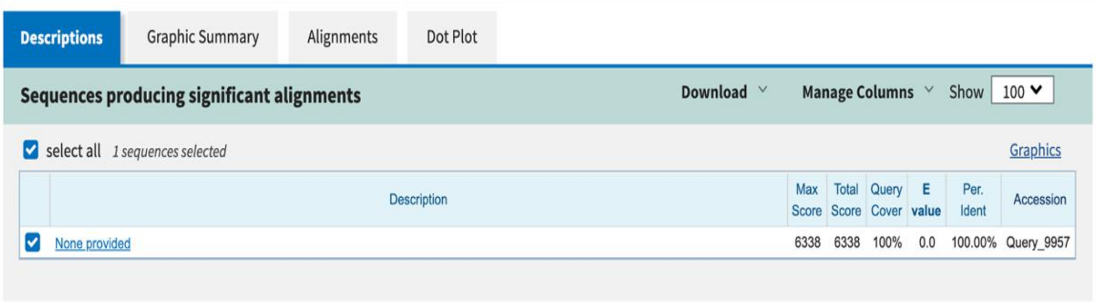
Descriptions
Graphic Summary
Alignments
Dot Plot
Sequences producing significant alignments
Download
Manage Columns
Show 100
select all 1 sequences selected
Graphics
Description
Max Score
Total Score
Query Cover
E value
Per. Ident
Accession
None provided
6338
6338
100%
0.0
100.00%
Query_9957

Fig. S2

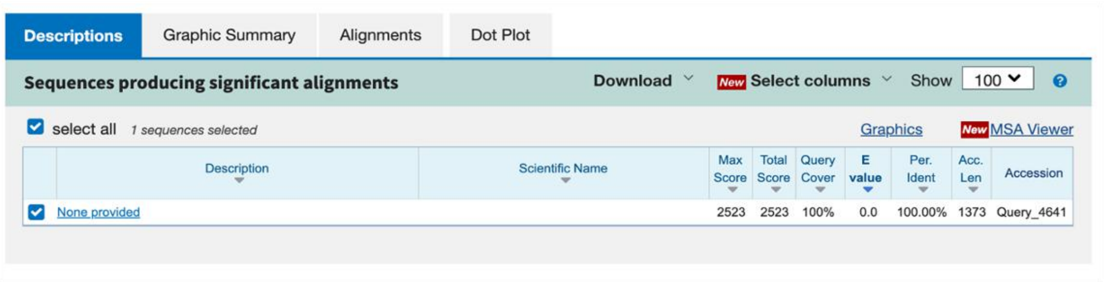
Descriptions | Graphic Summary | Alignments | Dot Plot

Sequences producing significant alignments   Download   New Select columns   Show 100

select all   1 sequences selected   Graphics   New MSA Viewer

	Description	Scientific Name	Max Score	Total Score	Query Cover	E value	Per. Ident	Acc. Len	Accession
☑	None provided		2523	2523	100%	0.0	100.00%	1373	Query_4641

Fig. S3

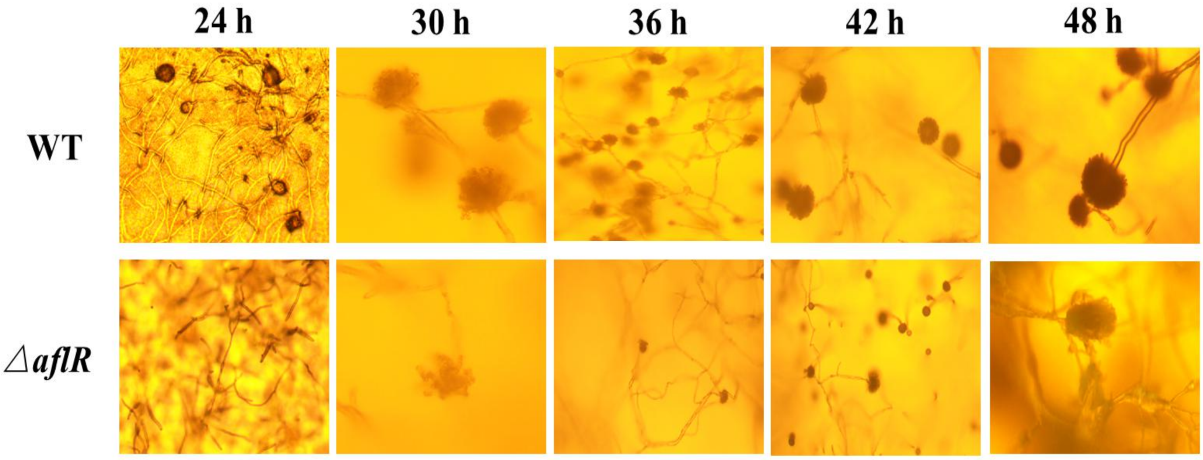

Fig. S4

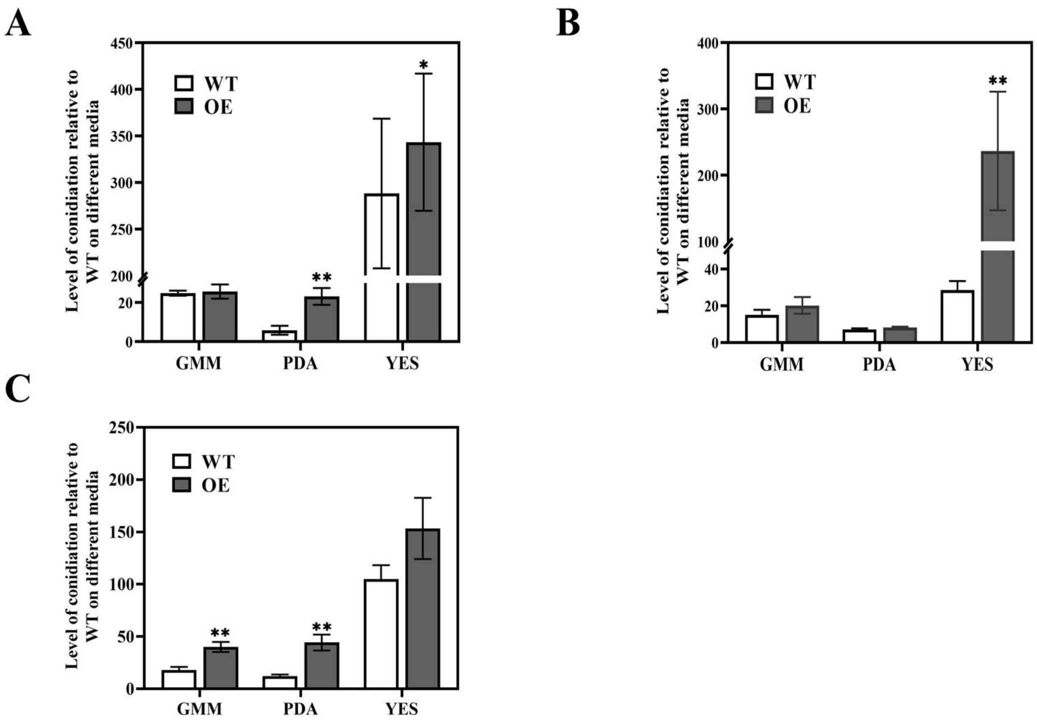
A
B
C
Level of conidiation relative to WT on different media
WT
OE
GMM
PDA
YES
0
20
200
250
300
350
400
450
0
20
40
100
200
300
400
0
50
100
150
200
250
*
**

Fig. S5

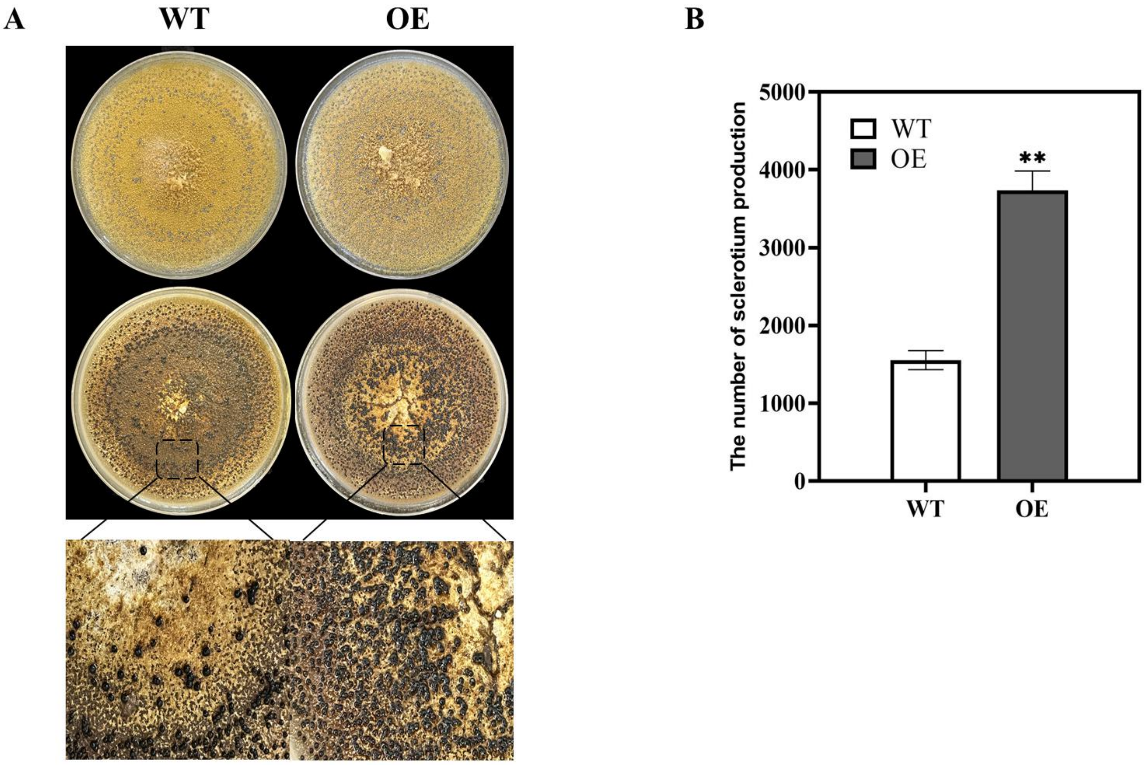
A
WT
OE
B
5000
4000
3000
2000
1000
0
WT
OE
**
The number of sclerotium production
WT
OE

Fig. S6

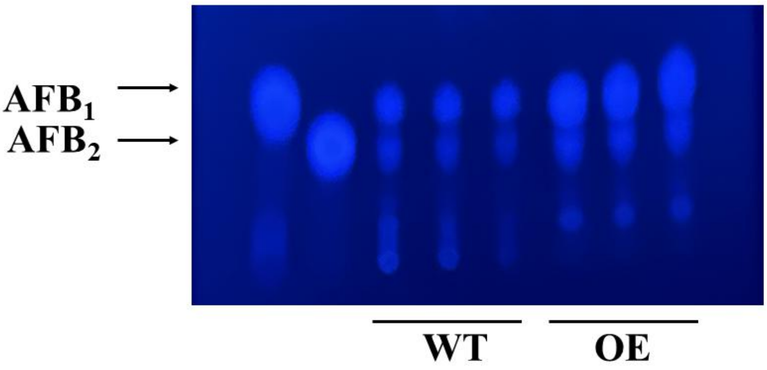

Fig. S7

Table S1 All primers used in the study

Primer	Sequence (5’-3’)
F5aflR880	CATTGGCAATCAGCGTTCG
R5aflR880	CAACATATTTCGTCAGACACAGAATAACTCTCCGTGGAGGTGAGGAAGGAATT
F3aflR1000	ATCAGTGCCTCCTCTCAGACAGAATAGAAAAGCCCCACCGC
R3aflR1000	GCATACCCGAAGTGTCAATCC
F5pyrG	GAGAGTTATTCTGTGTCTGACGAAATATGTTGT
R6pyrG	ATTCTGTCTGAGAGGAGGCACT
F5aflR880	CATTGGCAATCAGCGTTCG
R5check	TCTAAAATAGAAGCCGCCGG
F3check	GAGTGTTGTGGAGGAAGGCTG
R3aflR1000	GCATACCCGAAGTGTCAATCC
FtgaflR	ATGGTTGACCATATCTCCCCC
RtgaflR	TCATTCTCGATGCAGGTAATCAAT
FAflS	TTAATATCGGTTGTCATCGTTATCCAC
RAflS	ATGACCTTGACTGACCTAGAAACC
FpuC19	ATTACCTGCATCGAGAATGAAACTATGCGGCATCAGAGCAG
RpuC19	GCGAACGCTGATTGCCAATGGTCTGCTCCCGGCATCCGCTTACAGAC
OE F5aflR880	AGCGGATGCCGGGAGCAGACCATTGGCAATCAGCGTTCGC
OE R5aflR880	TCAGACACAGAATAACTCTCCGTGGAGGTGAGGAAGGAATTCAG
OE F5pyrG	ATTCCTTCCTCACCTCCACGGAGAGTTATTCTGTGTCTGACGAAATATGTTGT
OE R6pyrG	TGTCGTACCTTGGGAGCCATATTCTGTCTGAGAGGAGGCACTGA
OE FgpdA	TGCCTCCTCTCAGACAGAATATGGCTCCCAAGGTACGACA
OE RgpdA	GGGGAGATATGGTCAACCATTTGGGCATCAACCTTGGAGATGT
OE FtgaflR	TCTCCAAGGTTGATGCCCAAATGGTTGACCATATCTCCCCCC
OE RtgaflR	TGCTCTGATGCCGCATAGTTTCATTCTCGATGCAGGTAATCAATAATGTCG
